# New roles for nuclear EGFR in regulating the stability and translation of mRNAs associated with VEGF signaling

**DOI:** 10.1371/journal.pone.0189087

**Published:** 2017-12-18

**Authors:** Klaus Dittmann, Claus Mayer, Stefan Czemmel, Stephan M. Huber, H. Peter Rodemann

**Affiliations:** 1 Division of Radiobiology and Molecular Environmental Research, University of Tuebingen, Tuebingen, Germany; 2 Department of Radiation Oncology, University of Tuebingen, Tuebingen, Germany; 3 German Cancer Consortium (DKTK), partner site Tuebingen and German Cancer Research Center (DKFZ), Heidelberg, Germany; 4 Quantitative Biology Center (QBiC), University of Tuebingen, Tuebingen, Germany; Colorado State University, UNITED STATES

## Abstract

Cell membrane-associated epidermal growth factor receptor (EGFR) translocates into a perinuclear/nuclear location upon stimulation, where it complexes with mRNAs. Treatment with radiation and cisplatin decreases the amounts of mRNAs present within this complex. Gene array analyses of mRNAs in complex with immunoprecipitated nEGFR revealed significant enrichment of different mRNA species compared to the control immunoprecipitation. Functional annotation with help of DAVID Gene Ontology Analysis identified under other terms the HIF-1A/VEGF signaling pathway as one of the top scoring KEGG pathways. RT-PCR and western blots revealed the radiation-induced expression of mRNAs and proteins involved in HIF-1A/VEGF signaling. Simultaneously, the levels of the corresponding validated miRNAs within the complex containing nEGFR and mRNAs were decreased. This finding argues that an mRNA/miRNA/nEGFR complex regulates protein expression. Indeed, we detected the GW182, AGO2, PABPC1 and cNOT1 proteins, which belong to the deadenylase complex, in a complex with nuclear EGFR. Erlotinib-mediated inhibition of EGFR kinase reduced the radiation-induced increase in mRNA expression. In this context, erlotinib reduced AGO2 phosphorylation by the EGFR kinase at residue Y393, which was associated with increased cNOT1 deadenylase activity and reduced mRNA stability. To prove the roles of miRNAs in this context, we transfected cells with an inhibitor of Hsa-mir-1180p5, which targets the NFATC4 mRNA, an mRNA associated with VEGF signaling, or pretreated cells with erlotinib. Indeed, Hsa-mir-1180p5 knockdown increased and the erlotinib treatment decreased the expression of the NFATC4 protein. The expression of the NFATC4 protein controlled the cloning efficiency and radiosensitivity of A549 and FaDu tumor cells. Thus, this study is the first to show that a membrane-located tyrosine kinase receptor, such as EGFR, is internalized to a nuclear/perinuclear location upon exposure to stress and modulates the stability and translation of miRNA-selected mRNAs. This mechanism enables cells to directly express proteins in response to EGFR activation and may contribute to treatment resistance in EGFR-overexpressing tumors.

## Introduction

The receptor tyrosine kinase EGFR (ErbB1) plays a crucial role in both cancer initiation and progression [1–2] and is discussed as a promising target for cancer therapy. New insights into EGFR biology have established that EGFR signals through two distinct pathways: (i) canonical membrane-associated signaling [3–4] and (ii) non-canonical nuclear signaling to regulate gene expression, DNA replication and DNA damage repair [5–7].

The most intriguing step associated with nuclear EGFR signaling represents the physical translocation of the EGFR protein from the cell membrane to the perinuclear/nuclear location in response to cellular stress [7] or stimulation with EGF [8]. All four members of the ErbB family have been reported to undergo nuclear translocation [9]. Several distinct functions have been described for nuclear EGFR. Nuclear EGFR (nEGFR) is reported to act as a co-transcriptional activator for cyclin D1 [8, 10]. Furthermore, nEGFR controls proliferating cell nuclear antigen (PCNA) activity during DNA replication [11]. In the context of DNA repair, EGFR regulates DNA-PK activity [7, 12–14]. In addition, recent data [15] suggests a role for nEGFR in regulating mRNA stability and protein translation. Mechanistically, nEGFR regulates the ribonuclease activity of polynucleotide phosphorylase through DNA-PK-mediated phosphorylation [16], and consequently, the expression of the *c-myc* mRNA is increased. Moreover, the same study reported an inhibitory effect of EGFR-mediated argonaute 2 (AGO2) phosphorylation on miRNA maturation in response to hypoxia [17].

Importantly, miRNA-mediated RNA silencing represents an effective pathway to prevent the active translation of mRNAs. This process is performed by the RISC (RNA-induced silencing complex) in the perinuclear cytoplasm. Major elements of the RISC are AGO2 and GW182 (TNRC6), which are organized in protein complexes within the nucleus [18], where these RNAi factors are presumably involved in regulating gene transcription and mRNA splicing [19]. The GW182 protein acts as a platform that recruits and activates the deadenylase complex CCR4-NOT to the miRNA-directed target mRNA and facilitates the removal of the poly(A) tail. This process promotes mRNA degradation by AGO2 [19] and inhibition of translation [20]. Deadenylation is the major step triggering mRNA decay in eukaryotic cells [21]. The poly(A) tail and associated poly(A)-binding protein (PABP) interact with the 5’ m^7^G-cap/cap-binding complex to form a closed loop that enhances translation initiation and protects mRNA ends from nuclease attack [22]. Consequently, deadenylation represents an important control point for both mRNA degradation and translational silencing [21].

As shown in the present study, nEGFR is part of the miRNA-directed cNOT1 deadenylase complex and regulates the stability and translation of mRNAs through its kinase activity. In summary, we propose a mechanism by which nEGFR triggers a fast and efficient switch in protein translation in a miRNA-directed manner.

## Material and methods

### Cell culture and irradiation

Experiments were performed using the A549 human bronchial carcinoma cell line (ATCC CCL-185, Manassas, Virginia, USA), the FaDu head and neck tumor cell line (ATCC, HTB43), and HSF7 normal fibroblasts [23]. Irradiations were performed using the X-ray cabinet RS 225 (X-Strahl, Surrey, United Kingdom). The voltage was set to 200 kV, with a current of 15 mA (dose rate = 1 Gy/min at a 49 cm distance from the X-ray tube). The X-ray beam was hardened with a 0.5 mm removable copper filter. Dosimetry was performed with a farmer chamber (PTW, Freiburg, Germany). Irradiation was conducted at 37°C. Erlotinib was purchased from Selleck (Houston, Texas, USA) and EGF was purchased from Sigma-Aldrich (St. Louis, Missouri, USA).

### Subcellular protein fractionation

Cytoplasmic and nuclear extracts were prepared using the NE-PER^®^ kit (Thermo Scientific, Waltham, Massachusetts, USA), according to the manufacturer’s instructions.

### Quantification of mRNAs enriched in complexes containing nuclear EGFR

EGFR-IgG or IgG was covalently bound to an agarose support (direct IP-kit, Thermo Fisher Scientific/Pierce, #26148). Nuclear proteins were isolated from cells treated with the indicated compounds and EGFR complexes were precipitated by direct IP, in which the EGFR antibody (clone 13, BD Biosciences) was directly linked to agarose beads. We proved a lack of nonspecific complex formation by binding a nonspecific mouse IgG (isotype IgG1, Sigma) to agarose. Enriched mRNA templates were primed with oligo(dT)_12-18_ primer (Thermo Fisher Scientific) and transcribed into cDNAs using ImProm-II reverse transcriptase (Promega) in the presence of ^33^P-dATP. Free ^33^P-dATP was separated from labelled cDNAs using an Illustra NICK G50 Column (GE Healthcare). The incorporation of ^33^P-dATP in eluted cDNAs was quantified using a scintillation counter. Incorporated radioactivity was normalized to the nuclear EGFR protein concentration.

### Western blot analysis and immunoprecipitation

After irradiation, cells were lysed and proteins were resolved by SDS-PAGE. Western blotting was performed using standard procedures. All primary antibodies were diluted 1:1000 and purchased from the following sources: anti-EGFR (BD Transduction Laboratories, clone 1F4); anti-EGFR-pY992 (Abcam, ab81440), anti-lamin B1 (Biozol, clone ZL-5), anti-NFATC4 (Abcam, ab3447), anti-cNOT1 (Sigma, HPA046577), anti-GW182 (Biorbyt, orb183979), anti-AGO2 (Cell Signaling Technology, clone C34C6), anti-AGO2-pY393 (ECM Bioscience, AP5311), and anti-Actin (Sigma, A2066). Quantification was performed with the LI-COR detection system (LI-COR, Odyssey Fc). Immunoprecipitations were performed using the Pierce direct IP kit (#26148), according to the manufacturer’s instructions. Cell lysates were pre-cleared with nonspecific IgG bound to agarose beads.

### Expression of recEGFR-GST 3000–4000 containing the epitope for binding to the EGFR antibody clone 1F4

Total mRNAs were isolated from A549 cells and transcribed into cDNAs by RT-PCR. The GST coding sequence was amplified using the forward primer (Sall) 5`-ACGCGTCGACATAGTCGCCCAAAGTTCCGTGA-3` and the reverse primer (Not1) 5`- ATAAGAATGCGGCCGCATGCTACCAGCAAGCTTCTTCC-3´. After digestion at the restriction sites, the product was cloned in frame into the pGEX-4T vector (GE Healthcare) and transformed into competent *E*. *coli* cells. Positive colonies were selected and the proper orientation and correct reading frame were confirmed by sequencing. The expression of GST fusion protein and affinity purification were performed using standard procedures [24].

### DNA microarray analysis

A549 cells were irradiated with 4 Gy of radiation or sham irradiated, and nuclei were isolated from both samples 24 h after irradiation. Nuclei were lysed and EGFR or IgG immunoprecipitation was performed with a direct IP kit (Thermo-Fisher/Pierce, #26148). RNA was eluted from biological triplicates, transcribed into cDNAs, and hybridized to the Human Gene 2.1 ST Array (Affymetrix). The DNA microchip analysis was performed in cooperation with the Microarray Facility Tübingen (MFT, now c.ATG http://www.c-atg.de), and the statistical analysis was conducted in cooperation with the Quantitative Biology Center Tübingen (QBIC, https://portal.qbic.uni-tuebingen.de/portal/). Bioinformatics analyses of the microarray data were performed in the statistical language R (R version 3.1.1). For QC and data normalization, the R package ‘oligo’ (version 1.28.3) was used to quantitatively normalize probe intensities across all samples using the robust multi-array average (RMA) procedure [25]. The ‘genefilter’ package (version 1.46.1) was used to remove probes with very low variability between samples. Differential expression was analyzed using the package ‘Limma’ (version 3.20.9, [26]). For the Limma analysis, a linear model was fitted to the log2 expression data for each probe using the following formula: expr ~ construct * treatment. This model examines the effect of the factors construct (immunoprecipitation with EGFR versus immunoprecipitation with IgG) and treatment (non-irradiated versus irradiated) and their interaction on gene expression (expr). This model allowed us to extract coefficients/ratios for immunoprecipitation with nEGFR versus immunoprecipitation with IgG in cells that were not treated (contrast 1), the treatment effect for IgG (contrast 2) and the difference between contrast 1 and contrast 2 (the interaction term, contrast 3). Contrast 3, the interaction term in the model, equals the treatment effect for nEGFR. A post hoc interaction analysis was then performed with Limma and p-values were adjusted for multiple tests using [27]. For each of the three above-mentioned coefficients, gene expression was considered significantly different at an adjusted p value < 0.05. A log fold change cut-off was applied afterwards to only look at genes with a fold change > +1 or < -1. Raw data and metadata from the project were deposited into Gene Expression Omnibus (GEO) with the identifier GSE92428. Pathway analyses were performed using the DAVID functional annotation software [28].

### RT-PCR

A549 cells were pretreated with erlotinib (2 micromolar) for 2 h and subsequently irradiated with 4 Gy of radiation or sham irradiated. Twenty-four hours after irradiation, RNA was isolated from cells using an RNeasy Mini Kit (Qiagen). The cDNAs were generated with an RT2 First Strand Kit (Qiagen). Quantitative PCR was performed with the RT^2^ Profiler^™^ PCR Array for Human VEGF Signaling (Qiagen, #330231), according to the manufacturer’s instructions.

### Quantification of deadenylase activity

Nuclear fractions were isolated and the CCR4-NOT deadenylase complex was enriched by immunoprecipitation with a cNOT1 antibody, as described above. The assay was performed using a previously described method [29], with the following modifications. High-performance liquid chromatography-purified oligonucleotides were purchased from Eurofins. We used a 16-mer RNA substrate oligonucleotide (5’-CCU UUC CAA AAA AAA A-3’) containing a 5’-CY5 label, the 15-mer (Cy5-CCU UUC CAA AAA AAA-3’) and the 14-mer (Cy5-CCU UUC CAA AAA AA-3’ as a marker of deadenylation. In a standard reaction, cNOT1 precipitates were dissolved in 5 microliter of reaction buffer (20 mM Tris-HCl, pH 7.9, 50 mM NaCl, 2 mM MgCl_2_, 10% glycerol, and 1 mM beta-mercaptoethanol) containing 1.0 microliter RNA substrate in nuclease-free water. For gel-based detection and quantification of deadenylase activity, reactions were incubated at 30°C for 60 min, stopped by the addition of 12 microliter of RNA loading buffer (95% formamide, 0.025% bromophenol blue, 0.025% sodium dodecylsulfate and 5 mM ethylenediaminetetraacetic acid (EDTA)), and heated for 3 min at 85°C. A 3 microliter sample of the RNA mixture was analyzed by denaturing PAGE using a 20% acrylamide:bisacrylamide (19:1) gel containing 50% (w/v) urea. Intact mRNA labelled with the fluorescent dye Cy5 and deadenylation products were visualized and quantified using the LI-COR detection system.

### miRNA-mediated inhibition using the MISSION synthetic microRNA inhibitor

Inhibition of hsa-miR-1180-5p (NFATC4) was performed by transfecting A549 cells with the MISSION synthetic microRNA inhibitor (Sigma). Cells were seeded in 24-well plates at densities of 1 × 10^5^ cells in DMEM containing 10% FBS on the day before transfection. Cells were then transfected in triplicate with Lipofectamine 2000 and SYN MIRNA INHIB HUM hsa-mir-1180-5p (UUUCCGGCUCGCGUGGGUGUGU) or SYN MIRNA INHIB NEG. CONTROL 1 (GGUUCGUACGUACACUGUUCA) (25 nM). After 24 h, transfected cells were irradiated with 4 Gy of radiation, and after an additional 24 h, cell lysates were prepared for western blot analysis with an antibody against NFATC4.

### siRNA treatment

For EGFR or NFATC4 silencing, cells were treated with a specific siRNA for 72 hours before irradiation (ON-TARGETplus SMARTpool L-003114-00-0005 human EGFR, or ON-TARGETplus SMARTpool LQ-009584-00-0002, human NFATC4 siRNA, Dharmacon) and with on-TARGETplus Non-Targeting Pool siRNA (Dharmacon) as a control. Transfections were performed with Lipofectamine 2000 transfection reagent according to the manufacturer’s protocol (Invitrogen).

### Statistics

All data represent the means ± s.d. of three independent experiments. All statistical analyses, with the exception of the analysis of the microarray data, were performed using two-sample t-tests and Bonferroni’s correction for multiple testing. All raw data are given in supplementary file S1 File.

## Results

**Fig 1:**
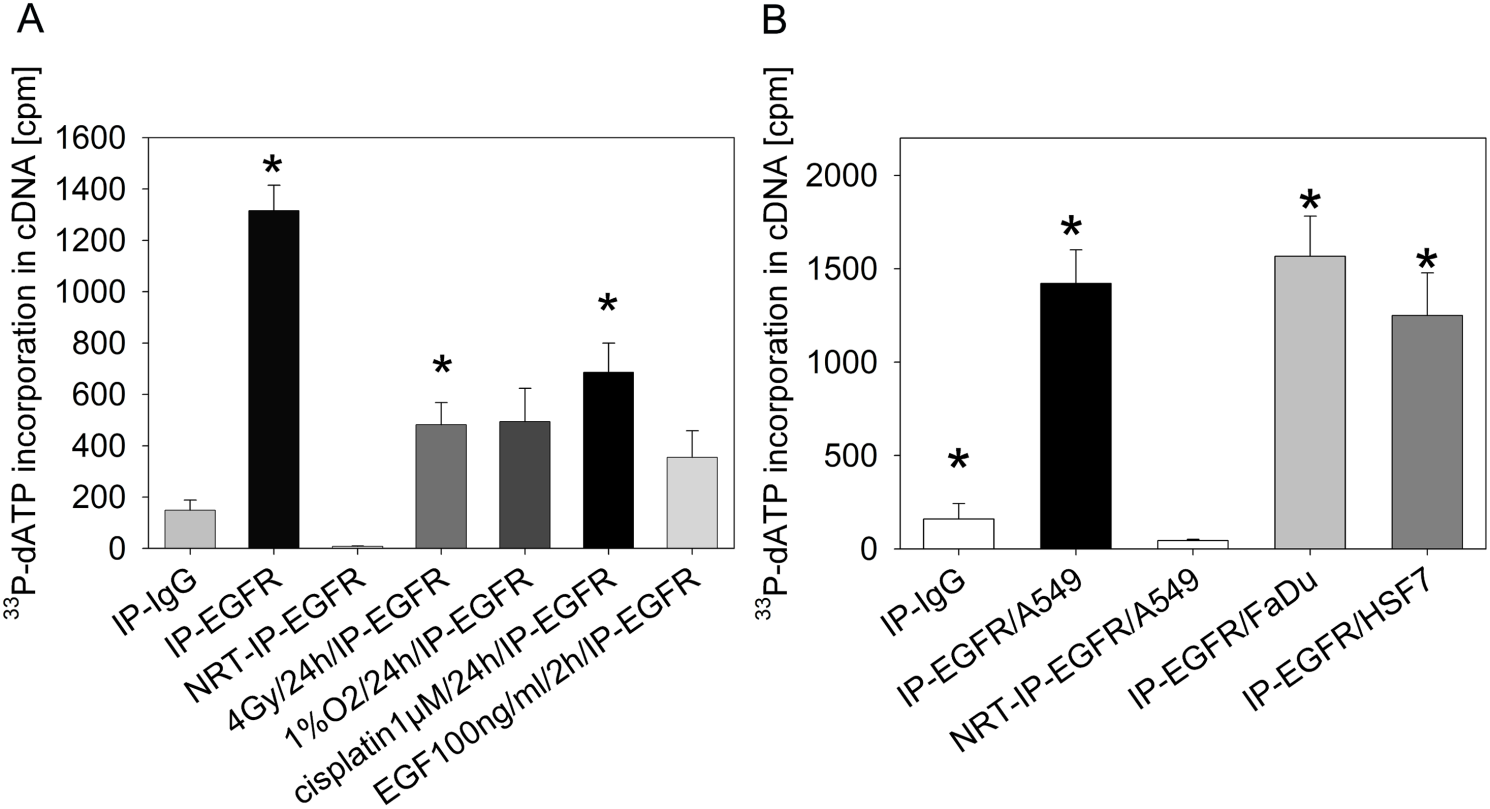
Detection of cDNAs synthesized from mRNAs enriched in complexes containing nuclear EGFR after exposure to different treatments in the presence of ^33^P-dATP. A: A549 cells were treated as indicated and EGFR was immunoprecipitated from the nuclear protein fraction (n = 3). The mRNAs that were bound to EGFR were reverse transcribed into cDNAs in the presence of ^33^P-dATP without amplification. Labeled cDNAs were isolated by gel filtration and the incorporated radioactivity was quantified using a scintillation counter. The detected radioactivity was normalized to nEGFR protein concentrations (S2A Fig). NRT = NRT control; we performed a mock reverse transcription reaction containing IP-EGFR and all RT-PCR reagents, with the exception of the reverse transcriptase. We performed a two-sample t-test combined with Bonferroni’s correction; *p < 0.008 compared to NRT-IP-EGFR. B: EGFR was immunoprecipitated from tumor cell lysates (A549 and FaDu) and normal skin fibroblast (HSF7) lysates. We performed a two-sample t-test combined with Bonferroni’s correction; *p < 0.01 compared to NRT-IP-EGFR/A549. Precipitates were used for reverse transcription in the presence of ^33^P-dATP. RNA was isolated by column chromatography and the amount of labelled mRNA was quantified using a scintillation counter. The incorporated radioactivity was normalized to nuclear EGFR protein concentrations (S2B Fig).

The observation that nEGFR is detected in a complex with mRNA binding proteins [15] suggests a potential role for nEGFR in mRNA processing. We synthesized cDNAs from mRNAs enriched in nEGFR complexes that were immunoprecipitated from nuclear extracts to elucidate the role of nEGFR in this process (Fig 1).

Immunoprecipitation with an anti-EGFR antibody enriched the EGFR protein in nuclear fractions (S1 Fig). The addition of a recEGFR-GST-fragment (68 kda) containing the epitope that binds the EGFR antibody reduced the precipitation of endogenous EGFR (180 kda), indicating the specificity of the applied antibody (S1 Fig). As described in a previous study [7], several cellular stress treatments increased the expression of the nuclear EGFR protein (S2A Fig).

Immunoprecipitates were used to transcribe mRNAs complexed with nuclear EGFR into cDNAs in a reaction containing poly (TT) primers and radioactive dATP. Free radioactivity was separated from labelled cDNAs by gel filtration and the incorporation of radioactively labelled dATP into cDNAs was quantified. After normalization to the concentration of the nuclear EGFR protein, the expression of mRNAs enriched in complexes with immunoprecipitated nEGFR was increased compared to the NRT-IP-EGFR-control (Fig 1A). However, irradiation and cisplatin treatments decreased the relative amount of enriched mRNAs complexed with nEGFR compared to untreated cells (Fig 1A). Notably, an immunoprecipitation with nonspecific IgG was also positive for mRNA enrichment–as presented in Fig 1B—, although to a much smaller extent. Enrichment of mRNAs in complex with nEGFR was not only observed in A549 cells but also in the FaDu head & neck tumor cell line and in HSF7 normal skin fibroblasts (Fig 1B).

**Fig 2:**
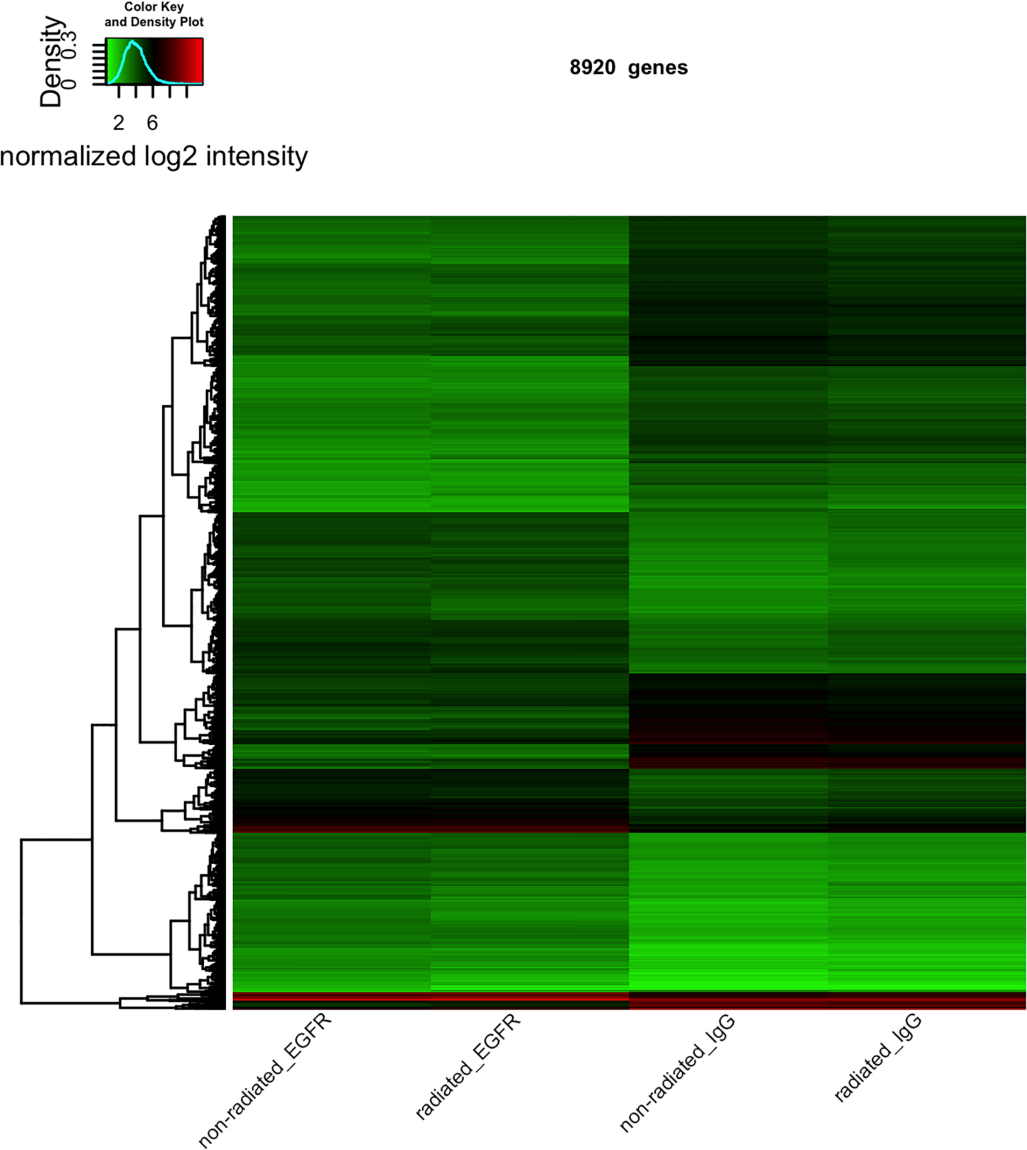
Heatmap-based visualization of the log2 normalized expression values. Heatmap displaying log2 expression ratios of the 8920 probes that were DE in non-irradiated cells that were immunoprecipitated with the EGFR antibody (first column) or IgG (third column)(p<0.05). We considered only oligos, which refer to genes and have in addition a log fold change either >+1 or < -1. Lower log2 ratios are indicated by a green color in the heatmap and higher log2 ratios are indicated by a red color. The dendrogram on the left side is based on Euclidean measures to obtain the distance matrix.

We performed an immunoprecipitation of nEGFR and the IgG control at time 0 without irradiation (0 Gy, non-irradiated) and also at 24 h with irradiation (4 Gy, irradiated) to elucidate the functional relevance of complexes between nEGFR and mRNAs. The mRNAs were extracted after immunoprecipitation from the complexes and characterized with the help of Affymetrix microarrays. After quality control of the microarray data, quantile normalization of the probe intensities was performed. Subsequently, normalized intensities were used to assess differential expression (DE) of genes and determine whether genes respond differently to immunoprecipitation with nEGFR versus immunoprecipitation with IgG in the absence of irradiation (contrast 1) or whether genes respond differently to radiation in IgG-precipitated samples (contrast 2). An interaction term was also added to the linear model (contrast 3, see the Materials and methods section) to determine whether a difference in gene expression was observed in nEGFR samples in response to radiation that differs from IgG samples. Following the DE analysis, we observed a strong effect on contrast 1, as 15068 probes of 53617 probes were DE (p < 0.05), when considering only oligos referring to genes. 8920 out of these 15068 probes have in addition a log fold change either > +1 or < -1 (S1_Tab.xls). No genes were DE with a multiple adjusted p value < 0.05 for contrast 2 and the interaction term (contrast 3, S2 Table). This finding indicates a strong influence of immunoprecipitation with either nEGFR or IgG on gene expression (contrast 1). However, a treatment/radiation effect that differed between IgG and EGFR samples was not observed (contrast 2 and 3). Heatmap-based visualization of the log2 normalized expression values (Fig 2) for the 8920 DE probes in contrast 1 revealed the differentially expressed genes when either immunoprecipitated with nEGFR (two left columns) or IgG (two right columns). The heatmap also indicated that most DE genes in contrast 1 were expressed at rather low levels (indicated by the green color in Fig 2).

We performed a functional annotation of all 8920 identified mRNAs in complex with nEGFR that were significant different in non-irradiated cells compared to IgG complexes (p<0.05) (S1 Table) using the DAVID Gene Ontology Analysis to further evaluate the binding of these mRNAs to nEGFR [28, 30]. DAVID matched 5515 of those mRNAs and the analysis resulted in the following top scoring KEGG pathway groups: Protein processing in the endoplasmic reticulum, Endocytosis, Proteasome, Lysosome, Ribosome, Cell cycle and HIF-1A signaling pathway (S3 Fig)

According to our previous study, nEGFR is involved in regulating HIF-1A and VEGF expression [15]; therefore, we focused in a first approach on mRNAs involved in HIF-1A /VEGF signaling. We isolated mRNAs from irradiated or non-irradiated A549 cells 24 h after irradiation and performed a quantitative RT-PCR to determine the expression of a panel of 84 mRNAs associated with HIF-1A /VEGF signaling in response to irradiation (Table 1). We correlated mRNA expression quantified by RT-PCR with constitutive enrichment of distinct mRNAs at nEGFR measured using an Affymetrix chip analysis (Table 1).

**Table 1:** Expression (RT-PCR) and binding (Affymetrix) of mRNAs associated with HIF-1A /VEGF signaling to nEGFR.

Symbol	Affymetrix EGFR-0 Gy log2 expression	RT-PCR fold change in response to 4 Gy	RT-PCR fold change in response to 4 Gy plus Erlotinib	Affymetrix rel. binding to EGFR after 4Gy
AKT1	2.3 ± 0.23	2.2 ± 0.3	1.1 ± 0.1	0.67 ± 0.1
AKT2	3.4 ± 0.4	1.59 ± 0.12	1.05 ± 0.05	0.7 ± 0.08
AKT3	3.6 ± 0.34	1.13 ± 0.09	0.9 ± 0.12	0.9 ± 0.1
ARNT	4.8 ± 0.4	1.58 ± 0.12	0.95 ± 0.1	0.98 ± 0.07
BAD	3.3 ± 0.34	2.62 ± 0.19	1.2 ± 0.3	0.8 ± 0.05
CASP9	3.6 ± 0.5	1.23 ± 0.13	1.1 ± 0.12	0.84 ± 0.04
CAV1	3.7 ± 0.4	1.94 ± 0.12	1.1 ± 0.13	0.69 ± 0.04
CDC42	3.1 ± 0.45	1.13 ± 0.2	0.9 ± 0.1	0.66 ± 0.03
FIGF	4.7 ± 0.6	1.0 ± 0.12	0.95 ± 0.1	0.85 ± 0.1
FLT1	4 ± 0.3	1.0 ± 0.2	1.0 ± 0.12	1.2 ± 0.07
FLT4	2.6 ± 0.34	1.94 ± 0.22	1.1 ± 0.12	0.83 ± 0.04
GRB2	3.6 ± 0.5	2.23 ± 0.12	1.3 ± 0.12	0.76 ± 0.05
HIF1A	3.4 ± 0.39	1.9 ± 0.11	0.83 ± 0.02	0.65 ± 0.08
HRAS	3.1 ± 0.23	1.9 ± 0.3	1.2 ± 0.15	0.8 ± 0.04
HSP90AA1	3.6 ± 0.2	1.0 ± 0.08	0.9 ± 0.09	0.67 ± 0.06
HSPB1	4.9 ± 0.45	3.16 ± 0.23	1.5 ± 0.19	0.58 ± 0.06
KDR		1.0 ± 0.02	1.1 ± 0.11	1.2 ± 0.06
KRAS	3.8 ± 0.6	1.17 ± 0.12	1 ± 0.15	1.1 ± 0.07
MAP2K1	5.3 ± 0.89	1.78 ± 0.3	0.8 ± 0.1	0.53 ± 0.09
MAP2K2	2.5 ± 0.76	3.71 ± 0.23	1.3 ± 0.3	0.63 ± 0.03
MAPK1	4.1 ± 0.53	1.45 ± 0.12	1.1 ± 0.1	0.61 ± 0.06
MAPK11	1.7 ± 0.23	2.1 ± 0.3	0.11 ± 0.05	0.74 ± 0.04
MAPK12	3 ± 0.54	2.48 ± 0.34	1.2 ± 0.16	1.1 ± 0.04
MAPK13	1.9 ± 0.2	1.8 ± 0.24	1.1 ± 0.12	0.82 ± 0.02
MAPK14	4.1 ± 0.23	1.54 ± 0.16	0.8 ± 0.13	0.79 ± 0.08
MAPK3	3.3 ± 0,34	1.73 ± 0.12	0.9 ± 0.17	0.64 ± 0,09
MAPKAPK2	2.8 ± 0.58	1.65 ± 0.13	0.75 ± 0.1	0.53 ± 0.07
MAPKAPK3	3.8 ± 0.69	1.91 ± 0.21	0.79 ± 0.02	0.83 ± 0.06
NFAT5	3.7 ± 0.56	1.18 ± 0,14	1.1 ± 0.1	0.67 ± 0.07
NFATC1	2.4 ± 0.43	1.46 ± 0.11	1.1 ± 0.1	0.88 ± 003
NFATC2	3.8 ± 0.51	1.0 ± 0.2	1.1 ± 0.12	1.07 ± 0.08
NFATC3	3.4 ± 0.87	1.38 ± 0.05	1.0 ± 0.05	0.82 ± 0.06
NFATC4	3.2 ± 0.45	3.32 ± 0.11	1.6 ± 0.19	0.79 ± 0.05
NOS3	3.3 ± 0.76	1.0 ± 0.05	1 ± 0.2	0.98 ± 0.05
NRAS	4.1 ± 0.92	1.28 ± 0.1	1.1 ± 0.1	0.73 ± 0.02
NRP1	5 ± 0.67	1.57 ± 0.11	1.1 ± 0.12	0.83 ± 0.05
NRP2	3.5 ± 0.34	1.87 ± 0.15	1.2 ± 0.2	0.82 ± 0.06
PDGFC	3.9 ± 0.29	1.47 ± 0.12	1.0 ± 0.1	1.05 ± 0.05
PGF	2.5 ± 0.34	5.62 ± 0.3	1.5 ± 0.4	1.1 ± 0.06
PIK3CA	4.1 ± 0.78	0.97 ± 0.05	1 ± 0.1	0.99 ± 0.06
PIK3CB	2.7 ± 0.12	1.26 ± 0.1	1.2 ± 0.09	0.75 ± 0.09
PIK3CD	2.4 ± 0.12	2.57 ± 0.29	1.5 ± 0.23	0.98 ± 0.07
PIK3CG	4.9 ± 0.34	1.0 ± 0.04	1.1 ± 0.1	1.24 ± 0.03
PIK3R1	4.2 ± 0.34	1.34 ± 0.16	1.1 ± 0.13	1.03 ± 0.05
PIK3R2	3 ± 0.64	2.36 ± 0.1	1,7 ± 0.05	0.78 ± 0.08
PIK3R3	3.4 ± 0.45	2.1 ± 0.1	1.5 ± 0.05	1.03 ± 0.05
PIK3R5	2.9 ± 0.87	1.0 ± 0.05	1.0± 0.2	0.98 ± 0.05
PLA2G10	4.5 ± 0.23	1.1 ± 0.1	1.1 ± 0.2	0.92 ± 0.05
PLA2G12A	4 ± 0.67	1.1 ± 0.2	1.0 ± 0.1	0.96 ± 0.07
PLA2G12B	3.7 ± 0.24	1.0 ± 0.08	0.8 ± 0.1	1.1 ± 0.04
PLA2G1B	4 ± 0.34	1.4 ± 0.1	0.8 ± 0.2	0.97 ± 0.08
PLA2G2A	2.7 ± 0.78	1.0 ± 0.03	0.9 ± 0.1	1.06 ± 0.08
PLA2G2D	3.5 ± 0.45	1.0 ± 0.08	1.0 ± 0.12	0.94 ± 0.04
PLA2G2E	2.7 ± 0.45	1.0 ± 0.03	1.1 ± 0.15	0.77 ± 0.06
PLA2G2F	2.6 ± 0.34	1.0 ± 0.07	1.0 ± 0.11	0.69 ± 0.05
PLA2G3	2.3 ± 0.34	1.0 ± 0.03	1.0 ± 0.12	0.86 ± 0.06
PLA2G4A	4.9 ± 0.45	1.28 ± 0.06	1.1 ± 0.11	0.77 ± 0.05
PLA2G4B	2.2 ± 0.45	4.29 ± 0.23	1.9 ± 0.3	0.77 ± 0.03
PLA2G5	3.8 ± 0.54	1.0 ± 0.04	1.1 ± 0.2	0.94 ± 0.05
PLA2G6	2.9 ± 0.43	2.16 ± 0.15	1.5 ± 0.1	0.88 ± 0.03
PLCG1	3.1 ± 0.23	2.73 ± 0.32	1.2 ± 0.15	0.66 ± 0.05
PLCG2	2.4 ± 0.12	1.65 ± 0.21	0.9 ± 0.1	0.97 ± 0.04
PPP3CA	3.7 ± 0.34	1.37 ± 0.19	1.1 ± 0.07	0.95 ± 0.04
PPP3CB	3.9 ± 0.54	1.22 ± 0.13	0.8 ± 0.1	1.0± 0.04
PPP3CC	3.6 ± 0.78	1.14 ± 0.11	0.6 ± 0.12	1.01 ± 0.07
PPP3R1	4.7 ± 0.98	1.58 ± 0.14	1.1 ± 0.17	1.1 ± 0.07
PPP3R2	3.1 ± 0.56	1.0 ± 0.05	0.9 ± 0.05	1.05 ± 0.07
PRKCA	4 ± 0.76	1.16 ± 0.19	0.8 ± 0.1	0.8 ± 0.03
PRKCB	3.4 ± 0.45	2.28 ± 0.3	1.1 ± 0.13	1.1 ± 0.03
PRKCG	3.1 ± 0.56	4.38 ± 0.34	1.9 ± 0.11	0.7 ± 0.04
PTGS2	4.1 ± 0.67	1.61 ±0,12	0.8 ± 0.1	0.65 ± 0.04
PTK2	3.3 ± 0.34	1.4 ± 0.12	1.1 ± 0.09	0.93 ± 0.04
PXN	2.9 ± 0.67	2.19 ± 0.18	1.2 ± 0.15	0.7 ± 005
RAC1	3.7 ± 0.56	0.81 ± 0.2	0.9 ± 0.14	0.73 ± 0.03
RAC2	3.9 ± 0.67	1.1 ± 0.15	1 ± 0.06	0.95 ± 0.04
RAF1	4.5 ± 0.87	1.87 ± 0.19	1.2 ± 0.1	0.83 ± 0.06
SH2D2A	3.1 ± 0.56	1.0 ± 0.02	1.1 ± 0.12	0.87 ± 0.04
SHC2	2.3 ± 0.45	1.07 ± 0.1	1.2 ± 0.2	0.72± 0.06
SPHK1	2.6 ± 0.67	3.01 ± 0.35	1.7 ± 0.12	0.89 ± 0.08
SPHK2	2.8 ± 0.28	2.16 ± 0.05	1.3 ± 0.18	0.7 ± 0.05
SRC	4 ± 1.2	1.82 ± 0.14	1.2 ± 0.09	0.7 ± 0.04
VEGFA	2.9 ± 0.93	1.18 ± 0.03	1.1 ± 0.09	0.82 ± 0.05
VEGFB	3.1 ± 0.45	2.16 ± 0.13	1.3 ± 0.08	0.98 ± 0.03
VEGFC	2.9 ± 0.34	1.01 ± 0.03	1.0 ± 0.13	1.04 ± 0.04
mean	3.6	1.68	1.1	0.86
SD	0.71	0.86	0.3	0.16
p =			0,0000000093	

Binding of mRNA to nEGFR and its relative change in response to irradiation is analyzed with help of Affymetrix gene chip analysis. Relative increase of mRNA expression in response to irradiation and its inhibition by Erlotinib treatment is shown as determined by RT-PCR.

Eighty-three of 84 mRNAs transcribed from the genes assigned to the RT^2^ Profiler^™^ PCR Array for Human VEGF/ HIF-1A -signaling (Qiagen) were detected in a constitutive complex with nEGFR in the absence of irradiation. We isolated mRNA from cells 24 h after irradiation and performed a quantitative RT-PCR for this panel of RNAs to elucidate the relevance of the formation of this complex. Fifty-three of the 84 mRNAs showed increased expression in response to irradiation (mean expression 1.68 ± 0.86) (Tab 1, column 2). Furthermore, treatment with the EGFR kinase inhibitor Erlotinib reduced the radiation-induced increase in the expression of 45 of 53 mRNAs (mean expression 1.1 ± 0.3, Table 1, column 3). Hence, we propose that nEGFR and its kinase activity are involved in regulating the increased expression/stability and translation of mRNAs associated with VEGF signaling in response to irradiation. We also determined the expression of representative proteins encoded by 10 of the 84 genes assigned to the RT^2^ Profiler^™^ PCR Array for Human VEGF/HIF-1A signaling to further elucidate the relevance of the formation of the complex between nEGFR and mRNAs. The increased mRNA expression observed in response to irradiation correlated with increased protein expression (S4 Fig). Moreover, we detected over 1000 different miRNA species in complex with nEGFR (GEO identifier GSE92428).

Notably, the amount of mRNA-specific miRNAs within the complex with nEGFR decreased in response to irradiation, whereas the amount of the corresponding mRNA and protein increased (S4 Fig).

**Fig 3:**
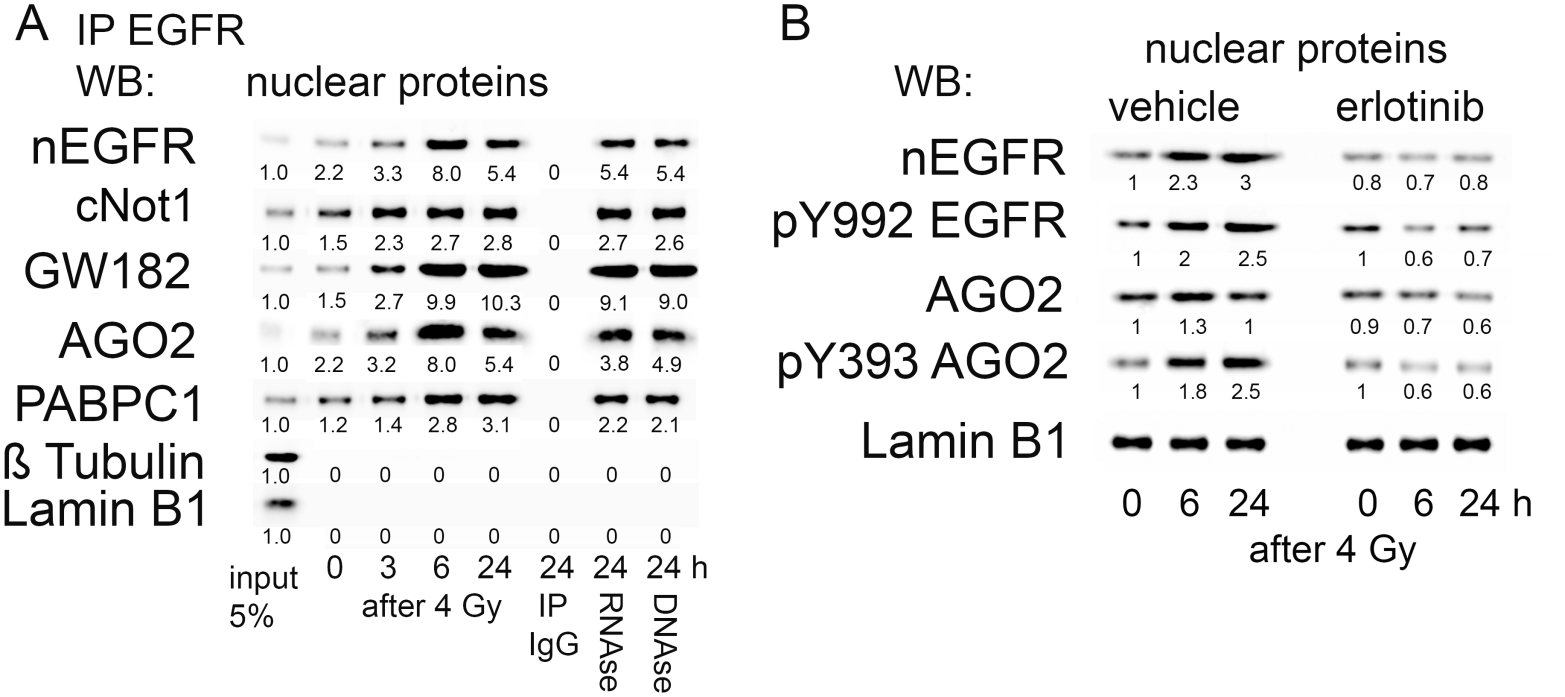
Interaction of nEGFR with members of the RISC. A: Immunoprecipitation of nEGFR in A549 cells. Detection of proteins involved in mRNA processing in complex with EGFR after irradiation with 4 Gy of radiation. Precipitates (24 h after IR) were treated either with 100 microgram/ml RNase A at 4°C for 1.5 h, or DNase I (50 U) for 30 min at 25°C. Beads were washed with PBS and denatured by adding protein sample buffer. Quantification of protein expression relative to input is shown. B: Inhibition of EGFR auto-phosphorylation and AGO2 phosphorylation after pretreatment with Erlotinib (2 micromolar/2 h) in the nuclear protein fraction of A549 cells. Representative results from three independent experiments are shown. We performed ANOVA followed by Tukey’s post hoc test to test for significant effects of Erlotinib on the data shown in Fig 3B. The effects of Erlotinib on nEGFR, pY992 EGFR, AGO2 and pY393 AGO2 expression were significantly different compared with the vehicle treatment (p<0.05).

Based on this observation, the net amount of mRNA-specific miRNAs in the complex with nEGFR negatively regulates the corresponding mRNA levels and protein translation. In summary, we hypothesize that nEGFR must be part of the P-bodies, which regulate mRNA translation and degradation in a miRNA-regulated manner [18]. Consequentially, we immunoprecipitated nEGFR and assessed the presence of the GW182 protein, a marker of RISC and P-bodies [31] (Fig 3A).

In addition to a radiation-induced increase in nEGFR expression, we observed the formation of a complex with the AGO2, PABPC1 and cNOT1 proteins, which all belong to the RNA-induced silencing complex (RISC) [31] (Fig 3A). The relative amounts of these proteins in the EGFR-complexes were approximately equivalent, suggesting that the radiation-induced increase in nEGFR expression is associated with increased formation of the complex with RISC proteins. None of these proteins were detected in the precipitate with the nonspecific IgGs. Neither the RNAse nor DNAse treatment resolved the protein complex, which argues for a direct protein interaction (Fig 3A). These observations strongly suggest the functional relevance of nEGFR in the context of regulating mRNA stability.

As further proof of the hypothesis that EGFR kinase is involved in AGO2 phosphorylation in response to irradiation, as has previously been reported for hypoxia [17], we detected increased amounts of AGO2 within the nuclear fraction, which was phosphorylated at residue Y393. Y393 phosphorylation is a well characterized phosphorylation event performed by EGFR kinase [17]. Importantly, blockade of EGFR kinase activity by pretreatment with erlotinib, as visualized by reduced auto-phosphorylation of nEGFR at Y992, reduced AGO2 phosphorylation at Y393 in response to irradiation (Fig 3B).

**Fig 4:**
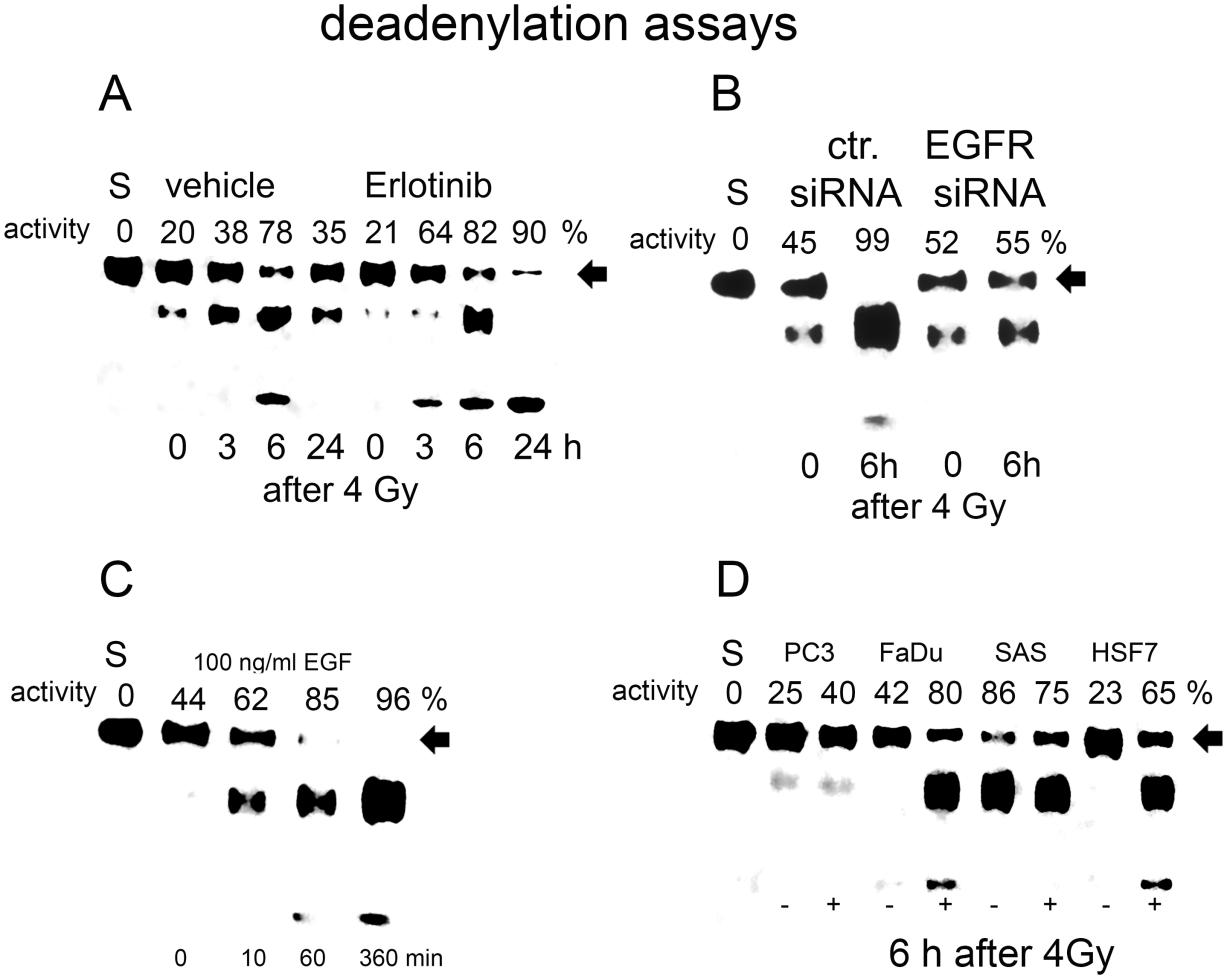
Quantification of cNOT1 adenylase activity after cNOT1 protein-IP. A: Activity was quantified after irradiation and erlotinib treatment. Activity inversely correlates with the residual amount of intact Cy3-labelled rec. mRNA substrate. Intact rec. mRNA (labelled with arrow) was quantified using LICOR gel quantification. Deadenylase activity was calculated from the amount of residual rec. mRNA compared to the amount of rec. mRNA used. S represents native rec. mRNA (0% activity). Data are presented as the means of 3 experiments. B: Quantification of radiation-induced cNOT1 deadenylase activity after EGFR knockdown with a specific siRNA. C: Quantification of cNOT1 deadenylase activity after EGF treatment. D: Quantification of cNOT1 deadenylase activity in different cell strains and lines 6 hours after irradiation.

The deadenylase cNOT1 is critically important in the regulation of mRNA stability [32]. The cNOT1 protein shortens the poly-A tails of mRNAs and initiates mRNA degradation. We applied an in vitro deadenylase assay to investigate the role of nEGFR in cNOT1 activity. We immunoprecipitated cNot1 from nuclear protein extracts and incubated it with a recombinant mRNA molecule terminally labelled with the Cy5 dye. Deadenylated products were separated by urea-PAGE gel electrophoresis and quantified using LICOR (Fig 4).

Deadenylation by cNOT1 was detected in the nuclear protein preparation, which included proteins from the perinuclear region. Irradiation increased deadenylase activity with a maximum at 6 hours and decreased 24 hours after irradiation (Fig 4A). Deadenylation was visualized by detecting smaller, deadenylated versions of the Cy3-labelled recombinant mRNA, and the quantified activity was inversely correlated with the amount of residual intact mRNA substrate. Bands were allocated to deadenylation products using specific markers, and cNOT1 antibody specificity was confirmed by incubating the substrate with a precipitate with nonspecific IgG (S5 Fig). The specificity of the deadenylation reaction was confirmed by knocking down cNot1 expression with a specific siRNA (S6 Fig).

Incubation of recRNA-polyA(8) with cNot1 immunoprecipitates for 1 h resulted in the appearance of a band corresponding to the first deadenylation product. This deadenylation reaction was blocked by the addition of poly A-oligonucleotides. Incubation of the intact recRNA-polyA(8) template with the control IP for 1 h did not produce a deadenylated product.

Pretreatment with erlotinib, which blocks EGFR kinase activity, strongly increased radiation-induced cNot1 activity (Fig 4A), whereas EGFR knockdown with a specific siRNA inhibited deadenylase activity (Fig 4B). Moreover, pretreatment of cells with EGF for 16 hours also induced cNot1 deadenylase activity (Fig 4C). Likewise, irradiation induced cNot1 deadenylase activity in several tumor cell lines, e.g., FaDu, SAS, and PC3 cells, and in normal fibroblasts (Fig 4D).

We transfected A549 cells with an inhibitor of a miRNA, hsa-miR1180-5p, which is enriched in the nEGFR complex and was validated to regulate stability and translation of the NFATC4 mRNA, to examine the hypothesis that nEGFR regulates mRNA stability and translation in concert with the cNot1 deadenylase and miRNAs. We selected this miRNA as a target, since hsa-miR1180-5p was the only miRNA within the nEGFR complex that targets the NFATC4 mRNA [33]. NFATC4 regulates HIF-1A/VEGF signaling and acts as a transcription factor that regulates cell survival, differentiation, angiogenesis, invasive migration, and the tumor microenvironment [34].

**Fig 5:**
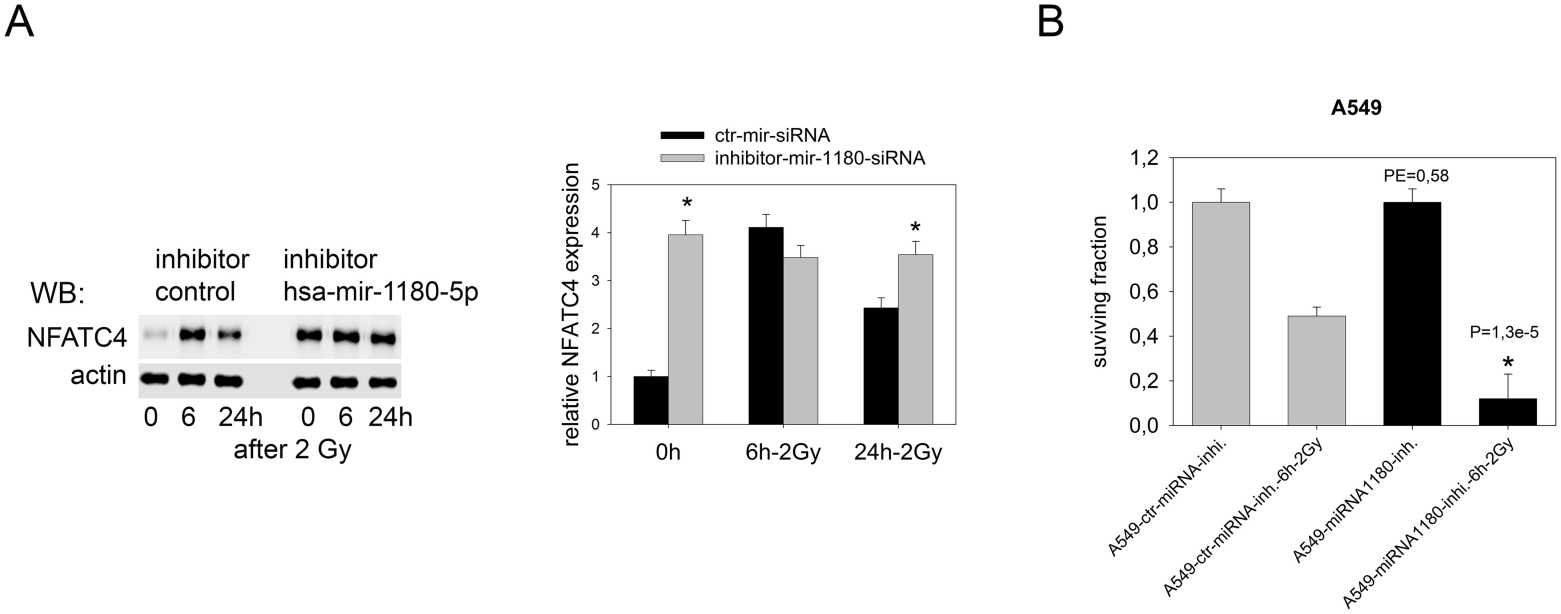
Role of hsa-mir-1180-5p in NFATC4 protein expression and function. A: Inhibition of radiation-induced downregulation of NFATC4 protein levels by blocking the effect of hsa-mir-1180-5p. A549 cells were treated with the hsa-mir-1180-5p inhibitor or inhibitor control for 24 h and irradiated with 2 Gy of radiation. Cells were lysed at the indicated time points and NFATC4 expression was quantified by western blotting. Densitometric quantification was performed on samples from three independent experiments (two-sample t-test combined with Bonferroni’s correction; *p< 0.016 compared to ctr-mir-siRNA). B: A colony formation assay was used to determine the effect of the hsa-mir-1180-p5 inhibitor on the plating efficiency and radio-sensitivity of A549 cells (*p< 0.016 compared to ctr mir-inhibitor).

Inhibition of hsa-mir1180-5p increased the expression of NFATC4 protein to a maximal level and prevented a further radiation-induced increase (Fig 5A). Since NFATC4 expression responds to irradiation (Table 1), we tested for a possible role for NFATC4 in the radiation response. Clonogenic survival after radiation treatment was determined to elucidate the effect of increased levels of the NFATC4 protein induced by hsa-mir1180-5p inhibition (Fig 5B). Increased NFATC4 protein expression reduced the plating efficiency and increased radiosensitivity of A549 cells. Comparable results for NFATC4 protein expression were achieved after EGFR expression was knocked down with a specific siRNA (S7 Fig). EGFR knockdown resulted in increased expression of the NFATC4 protein, which was accompanied by a lack of a radiation-induced increase in NFATC4 expression, similar to the control siRNA (S7 Fig).

**Fig 6:**
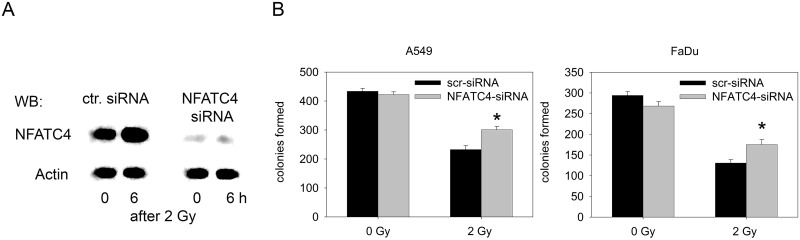
Inhibition of NFATC4 expression and its effect on clonogenic survival. A: Expression of the NFATC4 protein in A549 cells after irradiation and treatment with the NFATC-siRNA. B: Colony formation assay using A549 and FaDu cells after irradiation and treatment with the NFATC4 siRNA (two sample t-test; *p< 0.05 compared to scr-siRNA).

Since hsa-miR1180-5p does not exclusively target the NFATC4 mRNA, we directly knocked down NFATC4 expression with a specific siRNA (Fig 6). The knockdown of NFATC4 in A549 cells was efficient and protein expression was reduced (Fig 6A). This knockdown resulted in a radioprotective effect on both A549 and FaDu cells (Fig 6B).

## Discussion

Here, we described a mechanism by which nEGFR regulates the stability of mRNAs associated with VEGF signaling in a miRNA-directed manner. We immunoprecipitated EGFR from nuclear protein preparations and identified bound proteins using MALDI-TOF to elucidate the molecular function of nEGFR [15]. Under control conditions, nEGFR had already complexed with heterogeneous nuclear ribonucleoproteins (HNRNP) and PML bodies, which are involved in regulating gene transcription and mRNA translation during the cellular stress response [35–36], [36]. Indeed, based on published results from our group and other researchers, nuclear translocation of EGFR is induced by cellular stress, e.g., radiation [7], hypoxia [17], cisplatin [37], as well as its natural ligand, EGF [8]. Interestingly, the binding of mRNAs to EGFR was reduced significantly in response to radiation and cisplatin treatment. As shown in previous studies and the present study, nuclear EGFR translocation was accompanied by the activation of the tyrosine kinase [38]. Importantly, EGFR tyrosine kinase activity seemed to negatively correlate with mRNA enrichment. In addition, mRNA binding to nEGFR was observed not only in tumor cell lines but also in normal skin fibroblasts, suggesting a general role for nEGFR in regulating mRNA stability.

Using a microarray analysis, we were not able to identify a radiation-induced enrichment of mRNAs bound to nEGFR at 24 h that differed from IgG (control) in the present study. However, these results do not exclude the possibility that irradiation might alter mRNA binding to nEGFR compared to IgG at different time points. Although a treatment effect was not observed during the microarray analysis, we detected a significant enrichment of mRNAs in the EGFR complex compared to control IgG samples in the absence of stress treatment. Differential expression (DE) of 15068 of a total of 53617 mRNAs tested was detected. 8920 of them code for genes and have in addition a log fold change either > +1 or < -1 and were therefore estimated as potential biological relevant. We hypothesize that this large biological variation might only partially be related to the differential binding of mRNAs to nEGFR. Among other factors, nonspecific binding to nEGFR, genetic polymorphisms, and changes in mRNA levels due to age and genotype-environment interactions most likely also contribute to the observed high rate of differential expression [39].

The observation that mRNAs are also expressed in IgG-precipitated samples is consistent with previously published data [40] and also indicates that many tumor cells, including A549 cells, produce endogenous IgGs, which are involved in regulating tumor growth. The hypothesis that the list of DE genes contains specific and nonspecific signals is supported by gene ontology analysis, which pointed towards the regulation of terms such as cell cycle and ribosome. These categories are enriched in many experiments [41–42], indicating that these GO terms are not specific and do not need further interpretation. In contrast, the GO term HIF-1A/VEGF signaling pathway is potentially attributed to binding to nEGFR, as a link to nEGFR was observed in previously published data [15]. An RT-PCR analysis of 83 DE genes related to the HIF-1A/VEGF signaling pathway and the expression of proteins encoded by 10 selected genes from this group confirmed microarray data. Preliminary data also revealed a potential link between nuclear EGFR and mRNA stability, as the expression of genes involved in the HIF-1A/VEGF signaling pathway was slightly increased in response to irradiation, which was abrogated again by the EGFR kinase inhibitor erlotinib. However, based on the results from the microarray analysis, the radiation-induced increase in the expression of the candidate genes is not due to altered binding to nEGFR in response to irradiation. Therefore, we propose that additional factors play roles. In this context, a potentially important observation is that the radiation-induced increase in the expression of mRNAs associated with VEGF signaling and protein translation was associated with a simultaneous loss of miRNAs from the nEGFR complex. This loss was not significant for a single miRNA species, as determined by the array analysis. However, when we summed up the radiation-induced losses of different miRNAs validated to target specific mRNAs (up to 339 species per mRNA) from the nEGFR complex (S4 Fig), the loss was apparent. Nevertheless, the interplay between nEGFR, mRNAs and miRNAs has not yet been completely resolved yet. Our data indicate that nEGFR may participate in a central process that negatively regulates mRNA stability and translation in a miRNA dependent manner [43].

Based on these observations and the perinuclear location of EGFR in complex with mRNAs and miRNAs, we hypothesized that nEGFR is part of the P-bodies [18]. P-bodies are involved in mRNA storage and RISC-regulated mRNA degradation. Therefore, we assessed the localization of EGFR within P-bodies.

Indeed, we identified the RISC proteins cNot1, AGO2, GW182 and PABPC1 in complex with EGFR (Fig 3A). All proteins are located in P-bodies or GW-bodies in cells from higher eukaryotes and govern miRNA-mediated silencing to inhibit the active translation of mRNAs [18].

Translational repression of mRNAs is initiated by mRNA sorting to P-bodies for decapping-dependent decay or sequestration. Alternatively, active translation of mRNAs is inhibited by miRNA-mediated RNA silencing in the presence of GW182, the endonuclease AGO, and the CCR4-Not deadenylation complex [18]. However, although AGO 1–4 isoenzymes are capable of loading miRNA and exhibit endonuclease activity, RNAi-dependent gene silencing is exclusively executed by AGO2 (RISC) [44].

An interaction between AGO2 and nEGFR in response to hypoxia has been described [17]. Mechanistically, hypoxia activates EGFR kinase activity, which phosphorylates AGO2 at residue Y393, inhibits AGO2 activity and subsequently blocks miRNA maturation and loading to the miRNA-induced silencing complex (miRISC) [45]. Our data also suggest in addition to an effect on miRNA maturation, a role for the AGO2/EGFR interaction in the regulation of mRNA stability by the miRISC. This complex is a miRNA-recruited protein complex that regulates mRNA stability and subsequent protein translation [20]. The core of this complex consists of miRNA-loaded Argonaute and GW182 proteins [20]. In mammalian cells, RISC contains both poly(A)-binding proteins (PABP) and a deadenylase complex, which initiates the mRNA degradation process by inducing deadenylation [18, 46].

The mechanism by which protein translation is repressed is still not defined. The CCR4-Not protein complex recruited by the GW182 protein was recently shown to release PABP from the poly(A) tail, thereby disrupting mRNA circularization and facilitating translational repression and deadenylation [32].

As shown here, nEGFR complexes with GW182 and cNot1 proteins. Inhibition of EGFR kinase activity with erlotinib promoted cNot1 deadenylase activity. Thus, nEGFR kinase activity acts as a negative regulator of deadenylase activity. This idea agrees with the observation that nEGFR is a negative regulator of AGO2 in response to hypoxia [17]. In this context, nEGFR kinase activity is responsible for the phosphorylation of AGO2 at residue Tyr 393 in response to hypoxic stress, which suppresses dicer binding that is essential for microRNA maturation and RISC function.

The present data suggest an inhibitory function of AGO2 phosphorylation in the AGO2/GW182 complex, which regulates deadenylase activity [47–49]. On the other hand, downregulation of EGFR by a specific siRNA was associated with the absence of the EGFR protein in the nuclear fraction and the subsequent inhibition of cNot1 deadenylase activity. Thus, nEGFR is required for deadenylase activity. Moreover, the regulatory effect of nEGFR kinase activity on deadenylase activity was also observed after activation of EGFR kinase by EGF. We detected deadenylase activity in several tumor cell lines and in normal fibroblasts. Consequently, general roles for nEGFR kinase activity in regulating mRNA stability and protein translation are postulated.

After irradiation, the expression of mRNAs associated with VEGF signaling increased. Interestingly, pretreatment with erlotinib blocked the radiation-induced increase in mRNA expression. This finding supports the hypothesis that inhibition of EGFR kinase activity prevents AGO2 phosphorylation/inactivation and active cNot1-deadenylase degrades mRNAs located in the EGFR complex. The hypothesis that nEGFR is involved in regulating the expression of proteins involved in HIF1A/VEGF signaling is supported by previous studies showing that radiation-induced expression of the HIF-1A protein is reduced in response to erlotinib-mediated inhibition of nEGFR kinase activity [15].

The mRNA expression profile obtained from RT-PCR analysis of irradiated cells favors the increased expression of genes involved in angiogenesis and anaerobe glycolysis, which are markers of the metastatic tumor cell phenotype [50] in response to irradiation. In this context, EGFR is reported to be involved in regulating VEGFA expression in transformed cells [51] and human NSCLCs [52]. Moreover, as shown in our previous study, radiation induces HIF-1A expression to stimulate a metabolic shift toward lactate production, which is associated with treatment resistance [15]. We hypothesize that the mRNA/nEGFR complex may be relevant to radiation-induced tumor cell resistance.

According to the data presented in Table 1, the expression of mRNAs released from the complex with nEGFR was increased in response to irradiation. This finding may have resulted from mRNA stabilization and is consistent with the hypothesis that radiation-induced EGFR translocation and activation of kinase activity blocks AGO2 activity and deadenylase activity, subsequently resulting in mRNA stabilization. Importantly, we were able to identify validated miRNAs in complex with mRNAs and nEGFR. In response to irradiation these miRNAs were removed from nEGFR complex as AGO2 was phosphorylated and inactivated. These observations suggest the presence of a miRNA-driven deadenylase activity in complex with nEGFR. A miRNA-driven model for the regulation of cNot1 activity has been reported previously [31]. The present study postulates a novel role for nEGFR, which accumulates in perinuclear [7] and nuclear regions [15]. The nEGFR protein complexes with mRNA and proteins involved in the RISC and negatively regulates the deadenylase cNOT1 in a miRNA-directed manner through its kinase activity. Notably, EGFR kinase activity and nuclear translocation are induced by cellular stress. Moreover, this novel function of EGFR kinase is also observed in several EGF-stimulated tumor cell lines and normal skin fibroblasts.

The effect of hsa-mir1180-5p knockdown on NFATC4 protein expression links the presence of miRNAs in the EGFR/cNot1 complex with the translation of the corresponding protein. Furthermore, miRNA-regulated NFATC4 protein expression acts as a negative regulator of the post-radiation cell response and radiosensitivity. In general, NFATC4 acts as a negative regulator of growth and its expression is EGFR-dependent [53–54]. Consequently, as shown here for A549 and FaDu cells, knockdown of NFATC4 expression results in a radioprotective effect. These data suggest that NFATC4 controls cell survival in tumor cells after exposure to stress.

## Conclusions

In summary, the present study is the first time to show that nEGFR kinase-regulated cNOT1 deadenylase activity enables cells to immediately respond to cellular stress by interfering with mRNA stability in a miRNA-directed manner. We postulate a three-step regulatory mechanism. In the first step, mRNA species are loaded into a complex that contains nEGFR, GW182 and AGO2. The selectivity of this process has not yet been defined, but we assume that the regulation of the nuclear translocation of EGFR and constitutive formation of the complex with HNRN-proteins play roles in selectivity. In the second step, the deadenylase cNOT1 is loaded onto EGFR-bound mRNAs in a miRNA-guided manner. Third, deadenylase activity is regulated by EGFR kinase activity and determines mRNA stability and the translation frequency. Thus, a membrane bound tyrosine kinase receptor, such as EGFR, can directly increase the stability of mRNAs involved in regulating survival in a miRNA-dependent manner.

## Supporting information

S1 Fig Antibody effectiveness and specificity. EGFR was enriched from cell lysate and nuclear protein preparations (marker: beta-tubulin/lamin B1) using the EGFR antibody. Furthermore, we added a recombinant EGFR/GST-protein (recEGFR-GST 3000–4000) spanning the last 293 C-terminal amino acids of EGFR and containing the antibody epitope (aa 1020–1046) to nuclear lysates to suppress the binding of the antibody to cellular EGFR (180 KDa). Increasing concentrations of recEGFR (68 KDa) reduced the precipitation of native EGFR from nuclear lysate.
(TIF)

S2 Fig Fractionation and nuclear translocation of EGFR in response to different treatments. (A). A549 cells were treated as indicated for 1 h and cell lysates were then fractionated. Proteins were separated by SDS-PAGE and western blots were performed. B: Nuclear EGFR expression in the A549 and FaDu tumor cell lines and in HSF7 normal skin fibroblasts is shown.
(TIF)

S3 Fig Functional annotation. Functional annotation of mRNAs in complex with nEGFR using the DAVID Gene Ontology Analysis.
(TIF)

S4 Fig Protein expression, mRNA expression and validated miRNA in complex with the EGFR. Protein and mRNA expression of 10 of the genes assigned to the RT^2^ Profiler^™^ PCR Array for Human VEGF/HIF-1A-signaling in response to irradiation. The relative increase in expression in response to irradiation is shown. In addition, the simultaneous loss of all mRNA-specific miRNAs from complex with the nEGFR in response to irradiation is presented. Moreover, the numbers of mRNA-specific validated miRNAs present in the complex with EGFR is provided.
(TIF)

S5 Fig Deadenylation of the recRNA-polyA(8) template. Separation of recRNA-deadenylation markers: intact recRNA-polyA(8), recRNA-polyA(7), recRNA-polyA(6) and recRNA-polyA(0). Incubation of recRNA-polyA(0) with IP-cNot1 produced the same band as recRNA-polyA(0) alone.
(TIF)

S6 Fig Deadenylation of the recRNA-polyA(8) substrate after cNot1 knockdown. Incubation of the substrate with the cNot IP resulted in deadenylation. Pretreatment with the cNot1-siRNA blocked the deadenylation of the substrate. cNot1 knockdown was proven by western blotting. Knockdown was performed with ON-TARGETplus Human cNot1 siRNA (Dharmacon L-015369-01-0005) using standard procedures.
(TIF)

S7 Fig NFATC4 expression in response to irradiation and EGFR knockdown. A549 cells were treated with the EGFR siRNA or control siRNA for 24 h and irradiated with 4 Gy of radiation. Cells were lysed at the indicated time points and NFATC4 expression was quantified by western blotting. Densitometric quantification was performed on samples from three independent experiments.
(TIF)

S1 Table DE analysis for contrast 1. We considered only on oligos referring on genes. 8920 out of these 15068 probes have in addition a log fold change either > +1 or < -1.
(XLSX)

S2 Table DE analysis for contrast 3. We used a multiple adjusted p value < 0.05 for contrast 2 and the interaction term (contrast 3).
(XLSX)

S1 File Raw data. All raw data used are given in the folder S1_file.zip as Excel files.
(ZIP)
