# Supplementary figures and images for: New roles for nuclear EGFR in regulating the stability and translation of mRNAs associated with VEGF signaling
Source: PLoS One. 2017 Dec 18;12(12):e0189087. doi: 10.1371/journal.pone.0189087 (PMC5734708; doi:10.1371/journal.pone.0189087)

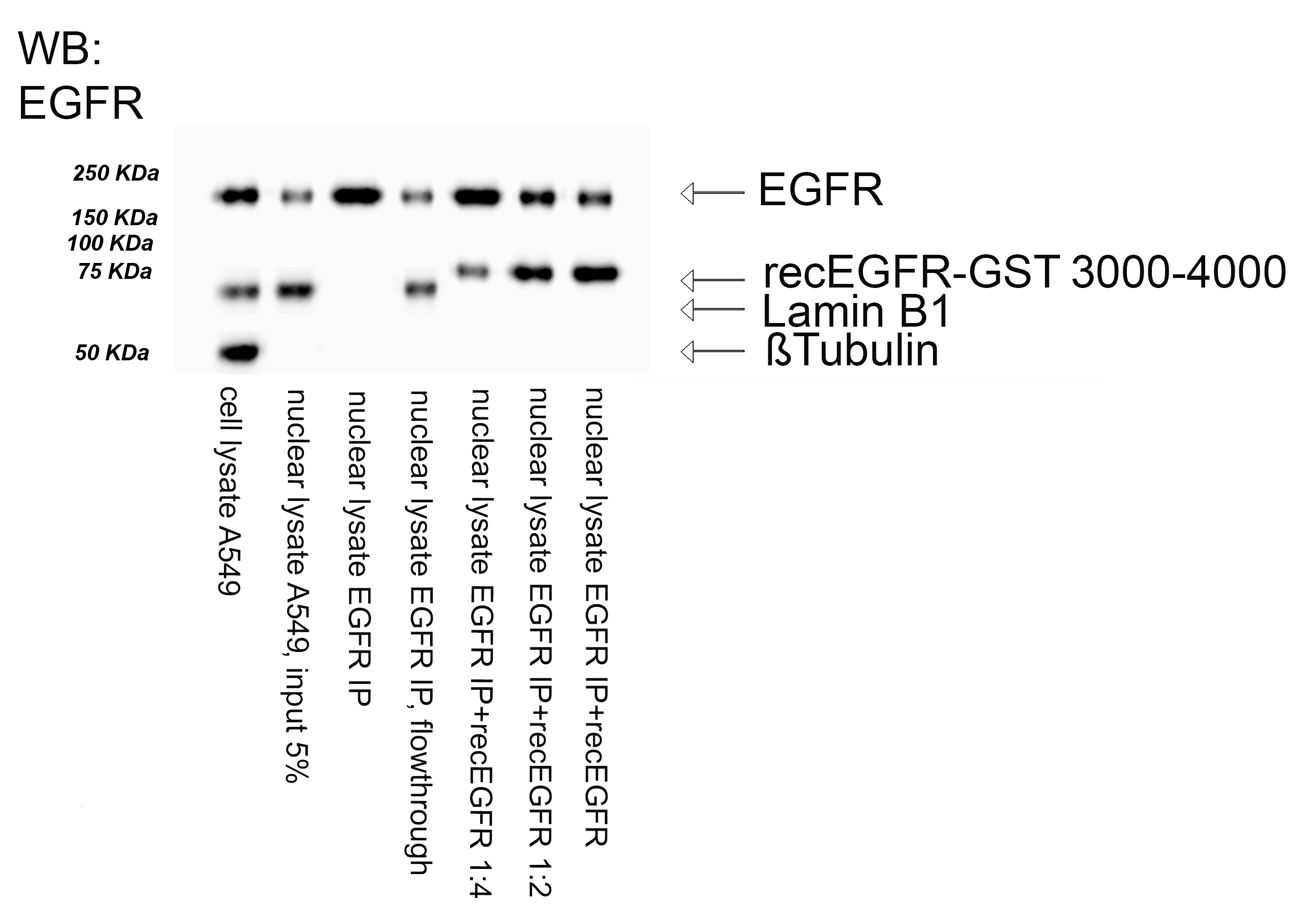

Supplement: S1 Fig — EGFR was enriched from cell lysate and nuclear protein preparations (marker: beta-tubulin/lamin B1) using the EGFR antibody. Furthermore, we added a recombinant EGFR/GST-protein (recEGFR-GST 3000–4000) spanning the last 293 C-terminal amino acids of EGFR and containing the antibody epitope (aa 1020–1046) to nuclear lysates to suppress the binding of the antibody to cellular EGFR (180 KDa). Increasing concentrations of recEGFR (68 KDa) reduced the precipitation of native EGFR from nuclear lysate. (TIF) [file pone.0189087.s001.tif]

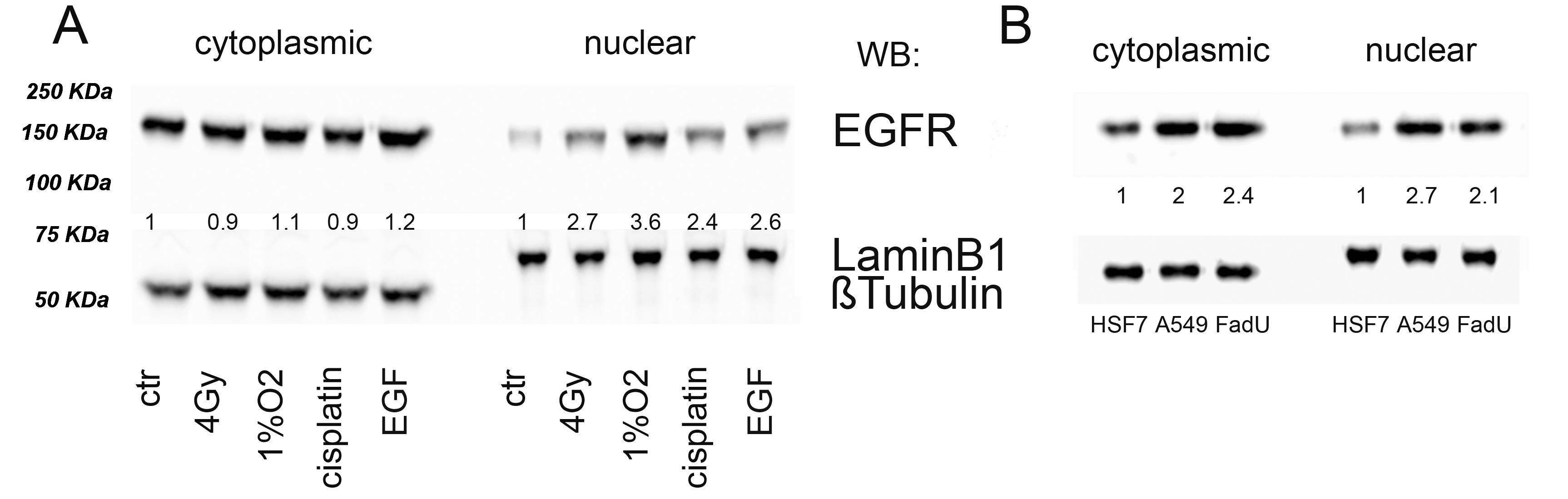

Supplement: S2 Fig — (A). A549 cells were treated as indicated for 1 h and cell lysates were then fractionated. Proteins were separated by SDS-PAGE and western blots were performed. B: Nuclear EGFR expression in the A549 and FaDu tumor cell lines and in HSF7 normal skin fibroblasts is shown. (TIF) [file pone.0189087.s002.tif]

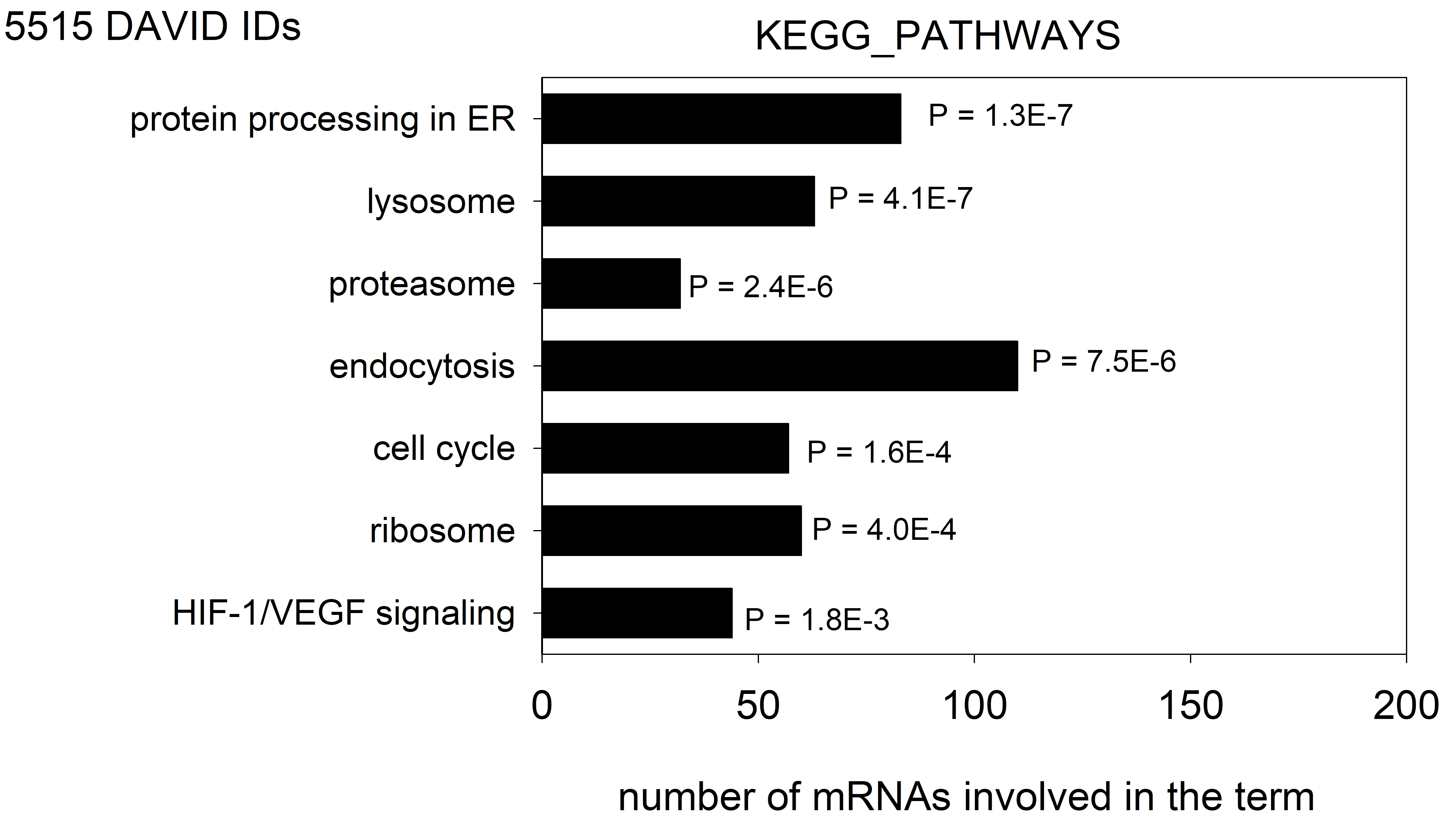

Supplement: S3 Fig — Functional annotation of mRNAs in complex with nEGFR using the DAVID Gene Ontology Analysis. (TIF) [file pone.0189087.s003.tif]

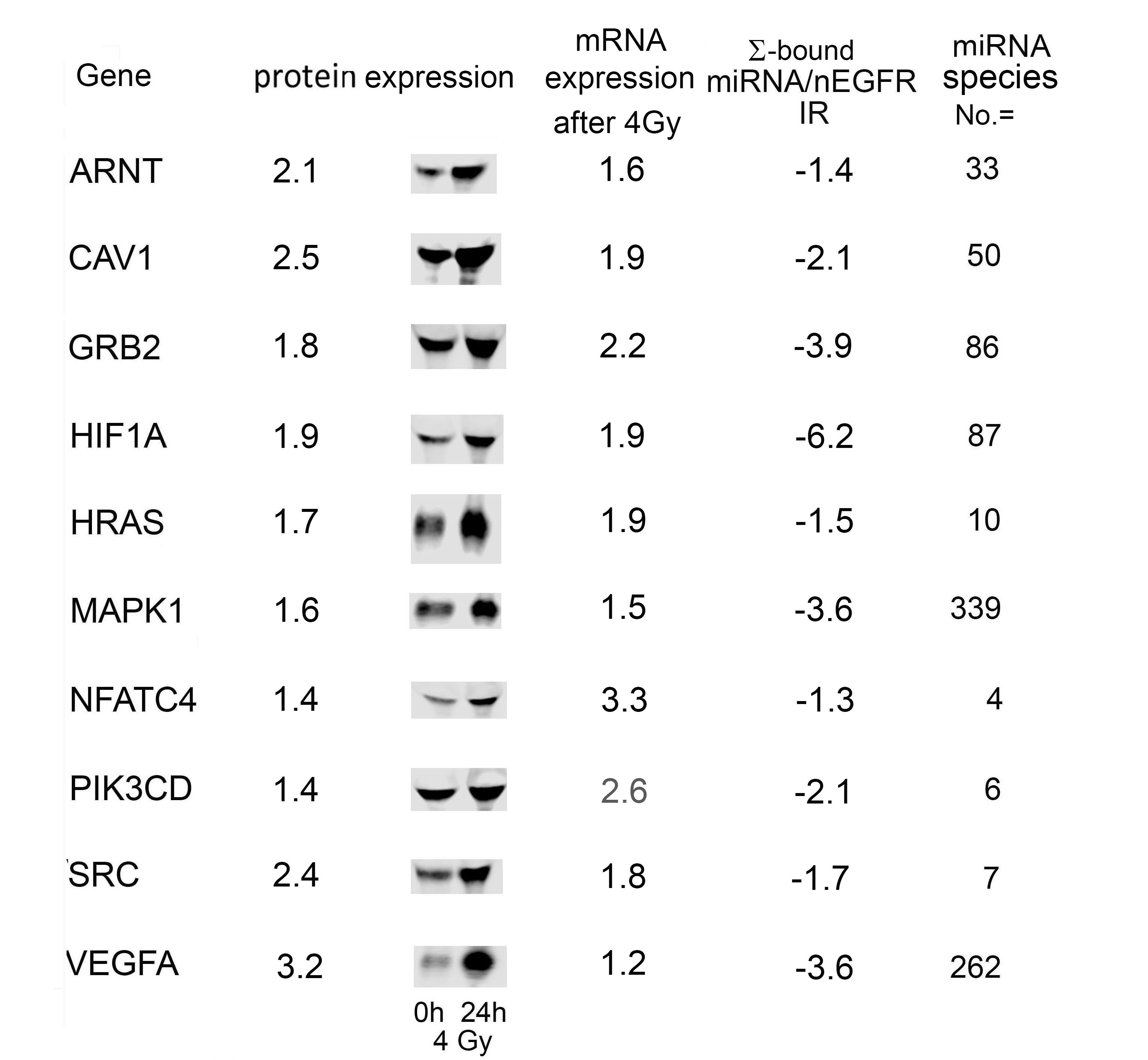

Supplement: S4 Fig — Protein and mRNA expression of 10 of the genes assigned to the RT2 Profiler™ PCR Array for Human VEGF/HIF-1A-signaling in response to irradiation. The relative increase in expression in response to irradiation is shown. In addition, the simultaneous loss of all mRNA-specific miRNAs from complex with the nEGFR in response to irradiation is presented. Moreover, the numbers of mRNA-specific validated miRNAs present in the complex with EGFR is provided. (TIF) [file pone.0189087.s004.tif]

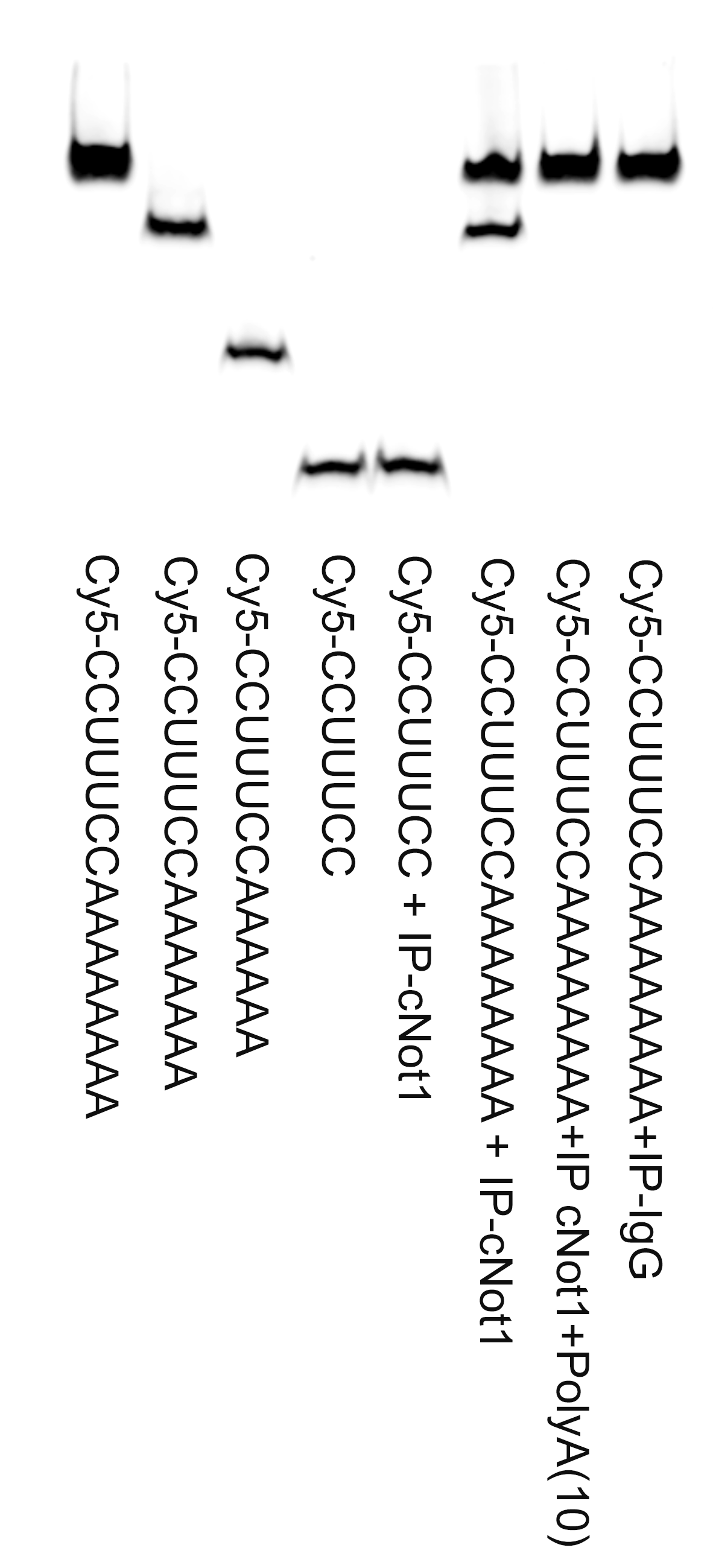

Supplement: S5 Fig — Separation of recRNA-deadenylation markers: intact recRNA-polyA(8), recRNA-polyA(7), recRNA-polyA(6) and recRNA-polyA(0). Incubation of recRNA-polyA(0) with IP-cNot1 produced the same band as recRNA-polyA(0) alone. (TIF) [file pone.0189087.s005.tif]

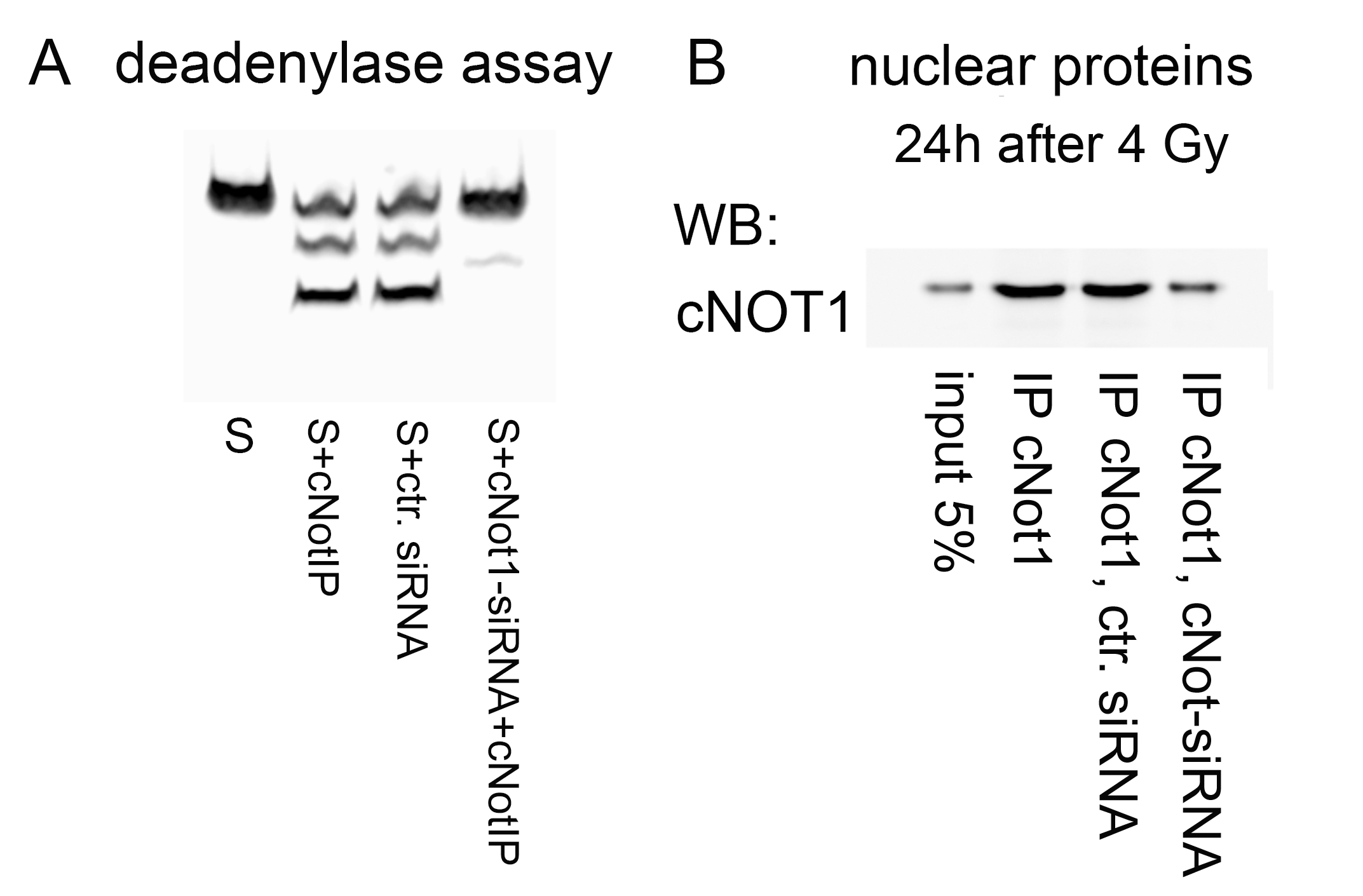

Supplement: S6 Fig — Incubation of the substrate with the cNot IP resulted in deadenylation. Pretreatment with the cNot1-siRNA blocked the deadenylation of the substrate. cNot1 knockdown was proven by western blotting. Knockdown was performed with ON-TARGETplus Human cNot1 siRNA (Dharmacon L-015369-01-0005) using standard procedures. (TIF) [file pone.0189087.s006.tif]

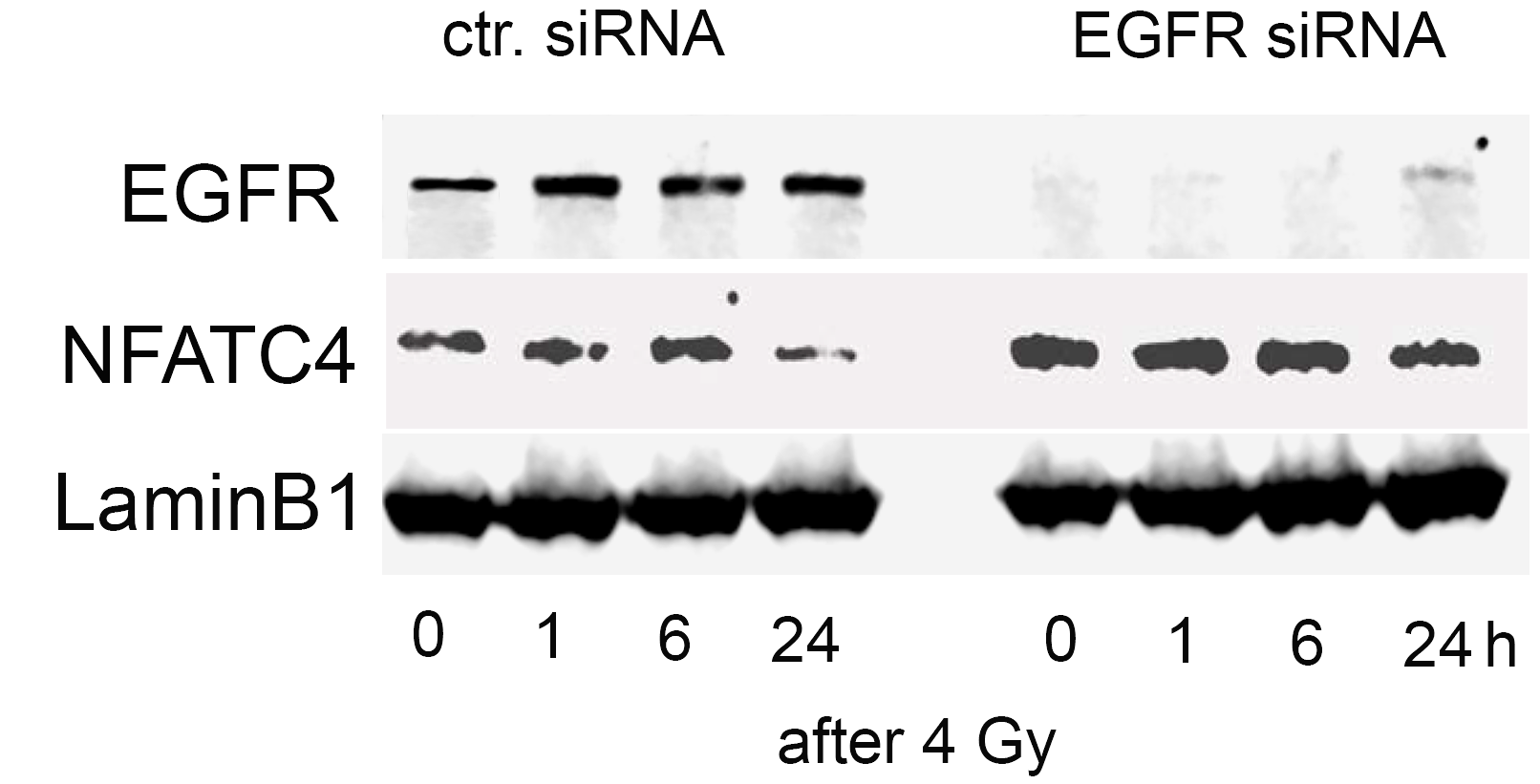

Supplement: S7 Fig — A549 cells were treated with the EGFR siRNA or control siRNA for 24 h and irradiated with 4 Gy of radiation. Cells were lysed at the indicated time points and NFATC4 expression was quantified by western blotting. Densitometric quantification was performed on samples from three independent experiments. (TIF) [file pone.0189087.s007.tif]
